# Post-Traumatic Stress Disorder and Atherosclerosis: Co-Phenomena, or Cause-and-Effect?

**DOI:** 10.1007/s11883-026-01467-3

**Published:** 2026-09-26

**Authors:** Mithil K. Penta, Saichandra Kalvakota, Robert T. Mallet

**Affiliations:** 1 Dept. Medical Education, University of North Texas Health Science Center, Fort Worth, TX 76107 USA; 2 Dept. Physiology and Anatomy, University of North Texas Health Science Center, 3500 Camp Bowie Blvd., Fort Worth, TX 76107 USA

**Keywords:** Angiotensin II, Atherosclerosis, Catecholamines, Inflammation, Limbic system, PTSD

## Abstract

**Purpose of Review:**

Post-traumatic stress disorder (PTSD), a trauma- and stressor-triggered psychiatric condition affecting approximately 10 million Americans, is a major risk factor for atherosclerosis and its devastating sequelae including ischemic heart disease, stroke and hypertension. PTSD and atherosclerosis share many pathogenic mechanisms impacting the limbic system, prefrontal cortex and systemic vasculature, including hyperactivation of the sympathetic and renin-angiotensin systems, inflammation, dysregulated coagulation and altered cortisol secretion. Whether PTSD is a direct cause of atherosclerosis, or an independent co-phenomenon, is an unresolved but pivotal question.

**Recent Findings:**

Recent evidence supports both sequential cause-and-effect, where PTSD-instigated mechanisms provoke and/or perpetuate atherosclerosis, and parallel development of both phenomena from shared trauma-activated mechanisms. Mounting evidence also supports a bidirectional interaction, wherein the cardiovascular consequences of atherosclerosis elicit chronic psychiatric stress and, potentially, a maladaptive feedforward PTSD – atherosclerosis cycle. The specific pathobiological context may determine the prevailing relationship.

**Summary:**

Recent research has revealed multifaceted interactions among the neuroendocrine cascades mediating PTSD and atherosclerosis, and demonstrated complex associations between the two disorders. Knowledge of the PTSD – atherosclerosis relationship in the individual patient will inform efforts to optimize treatment of these complicated conditions.

**Graphical Abstract:**

**Figure:**
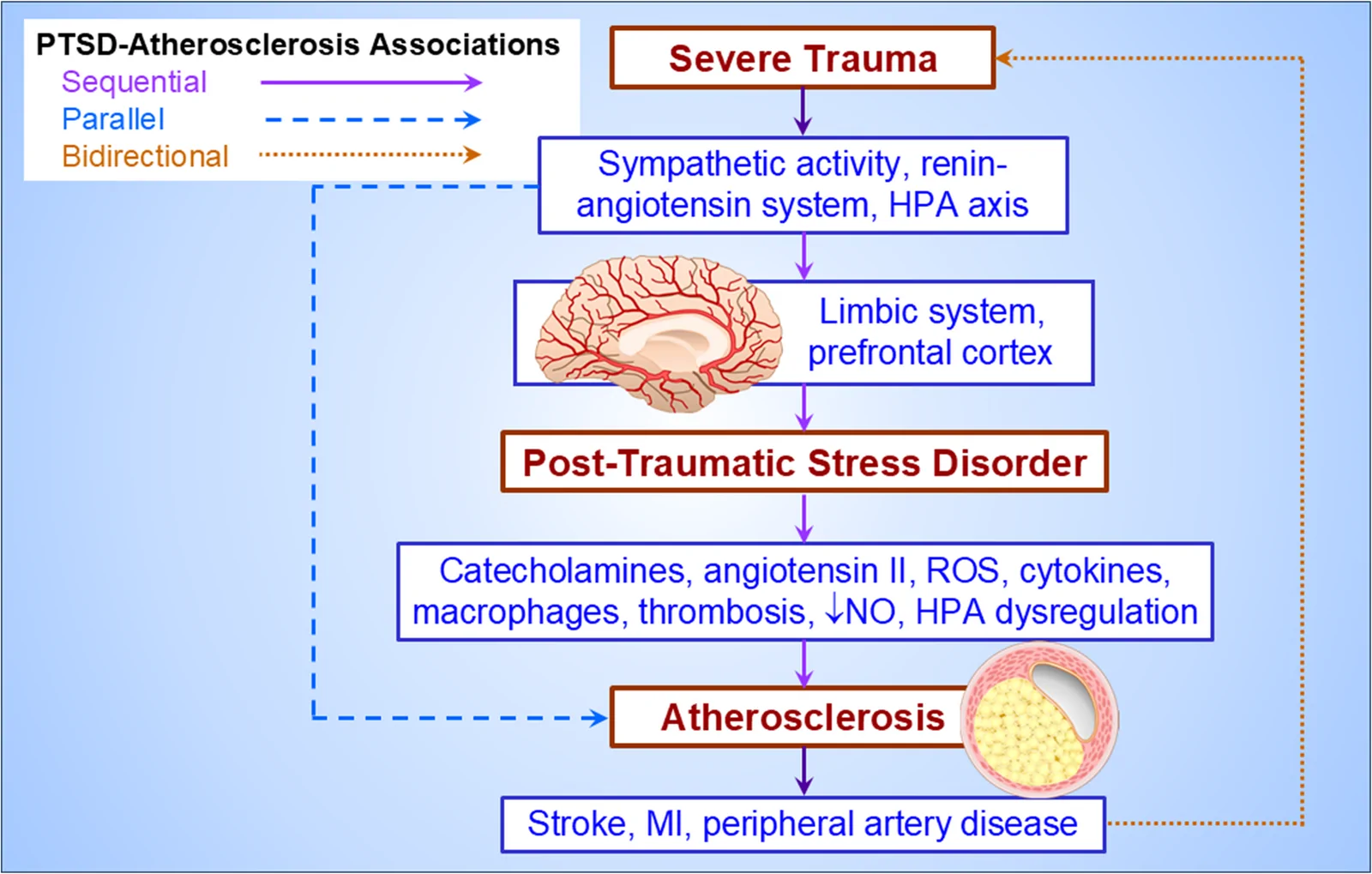

## Introduction

 Post-traumatic stress disorder (PTSD), a chronic, severe psychiatric condition that develops after exposure to one or more exceptionally traumatic events [1], is a major risk factor for atherosclerosis and its cardiovascular sequelae [2, 3]. Clinical studies have established associations between PTSD and measures of atherosclerosis including carotid intima-media thickness, arterial plaque accumulation, endothelial dysfunction and coronary artery calcification [4]. Adverse psychosocial conditions are established risk factors for cardiovascular diseases, particularly atherosclerosis impacting conduit arteries [5] including the aorta [6], coronary arteries [4, 7] and carotid arteries [6, 8]. Pathobiological signals linking psychiatric stress and atherosclerosis include autonomic imbalance, abnormal cortisol release, inflammation and prothrombotic states, underscoring the potential for PTSD-associated neuroendocrine dysfunction to provoke atherogenesis.

However, whether the PTSD – atherosclerosis relationship represents true causation vs. parallel phenomena remains uncertain. The evidence is equivocal: some studies support causality, while in others, adjusting for confounders including familial, cardiovascular or environmental risk factors weakens the independent relationship [6, 9, 10]. This article discusses recent preclinical and clinical evidence bearing on the complex nature of the PTSD – atherosclerosis relationship, including the potential for PTSD induction by atherosclerotic cardiovascular disease, and the interplay of the myriad mechanisms potentially linking the two phenomena.

## Methodology

Original research articles, reviews and meta-analyses pertinent to this review were identified by applying the following keyword searches to the U.S. National Center for Biotechnology Information’s PubMed^®^ database from June-August 2026: ‘PTSD’ AND ‘atherosclerosis’ [37 results, 2002–2026]; ‘atherosclerosis’ AND (‘chronic stress’ OR ‘sympathetic hyperactivity’ OR ‘hyperadrenergic’) [202 results, 1993–2026]; ‘PTSD’ AND (‘angiotensin II’ OR ‘hypercoagulability’) [45 results, 2003–2026]; (‘PTSD’ OR ‘chronic stress’) AND (‘NOS’ OR ‘NO stores’) [103 results, 1996–2026]; ‘PTSD’ AND (‘amygdala’ OR ‘hippocampus’) AND (‘hypertrophy’ OR ‘atrophy’ OR ‘apoptosis’) [148 results, 1996–2026]; and ‘atherosclerosis’ AND ‘endothelium’ AND (‘atheroma’ OR ‘plaque’) AND (‘macrophage’ OR ‘foam cell’) AND (‘inflammation’ OR ‘cytokines’) [378 results, 1985–2026]. Collectively, these searches identified 876 distinct articles. When the article abstract demonstrated alignment with the scope of this review, the full article was examined for possible citation. Priority was assigned to peer-reviewed articles reporting findings in humans and vertebrate animal models of PTSD and atherosclerosis. Unreviewed preprints were not considered.

## Post-Traumatic Stress Disorder: Diagnostic Hallmarks and Etiology

The PTSD diagnostic criteria include heightened anxiety, trauma-associated adverse alterations in cognition and mood, exaggerated startle reflex, hyperarousal and reactivity, nightmares and disordered sleep, occurrence of intrusive memories, physiological reactions to trauma-recapitulating cues, and avoidance of trauma-associated stimuli [1]. PTSD follows variable clinical trajectories [11]: although the first symptoms typically appear within 3 months of the traumatic event, in 20–30% of cases the constellation of symptoms fulfilling the PTSD diagnostic criteria are delayed a year or more [12, 13]. Failure to achieve timely resolution of the stressor and/or the resultant neuropathological responses contribute to PTSD pathogenesis and persistence [11–13].

The lifetime PTSD prevalence in the United States is 6–7% [14], with the highest rates in survivors or witnesses of sexual violence, military combat, natural disasters, and adverse medical events. Military veterans have a lifetime PTSD prevalence of 12.6–16.4% [15], and 12–40% of paramedics met the *DSM-5* criteria for PTSD diagnosis [1] in 6 cross-sectional clinical trials [16]. PTSD prevalence is approximately threefold greater in women and adolescent females than their male counterparts [17].

Traumatic stress provokes myriad maladaptive responses in the brain. Disruption of resting state functional connectivity within fear processing and memory formation networks predisposes to PTSD [18]. Centrally, PTSD primarily impacts the limbic system, particularly the amygdala and hippocampus, and the ventromedial prefrontal cortex [19]. In patients with major depressive disorder, comorbid PTSD was associated with hippocampal atrophy, thinning of the anterior cingulate cortex and hyperconnectivity of motor, sensory and cerebellar-prefrontal circuits [20]. In PTSD, the amygdala is hyperactive and, in rodent PTSD models, its neurons hypertrophy [21], while the hippocampal cornu ammonis 3 and dentate gyrus subfields atrophy [22] and undergo neuronal apoptosis [23, 24] and dendritic debranching [21, 25].

**Fig. 1:**
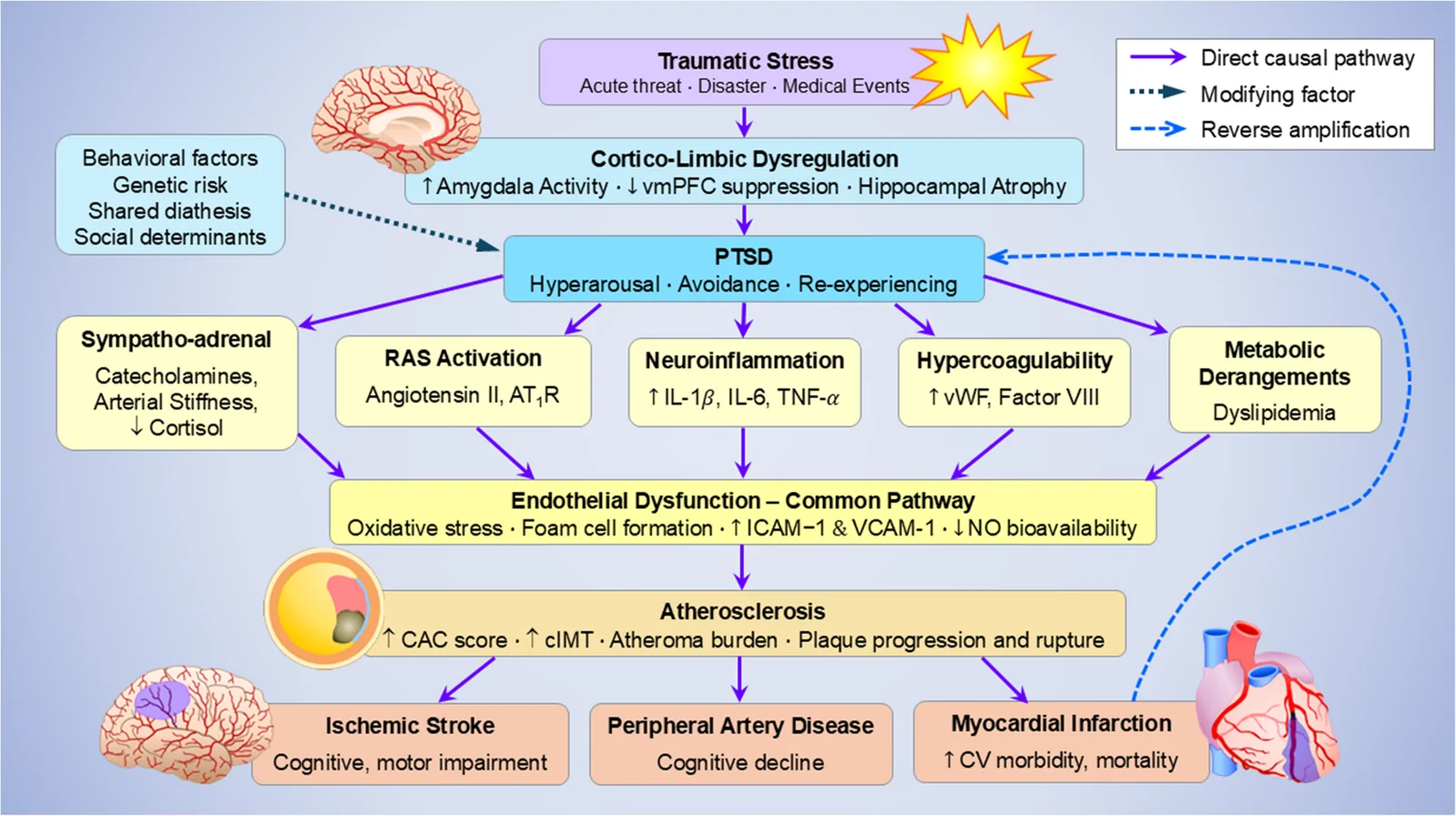
Sequential trauma – PTSD – atherosclerosis cascade and neuroendocrine connectors. Acute episodes of severe trauma hyperactivate the amygdala, suppress hippocampal neurons and disrupt the cortico-amygdala tracts, allowing chronic activation of the ventral prefrontal cortex culminating in post-traumatic stress disorder (PTSD). PTSD hyperactivates the adrenergic and renin-angiotensin (RAS) systems, inflammatory and coagulation cascades and hyperlipidemia, the PTSD-atherosclerosis connectors which collectively derange endothelial function, transform macrophages and vascular smooth muscle into lipid-engorged foam cells, provoke adhesion molecule expression and impair endothelial production of the anti-inflammatory vasodilator, nitric oxide (NO). Atherosclerosis ensues: coronary artery calcification (CAC), intima-media thickening (IMT) and atheroma expansion narrow the lumina of conduit arteries. Atherosclerosis heightens risk of ischemic stroke, cerebral hypoperfusion and impaired cognitions, and ischemic heart disease. AT_1_R: angiotensin type 1 receptor; cIMT: carotid IMT; ICAM-1: intercellular adhesion molecule-1; IL: interleukin; TNF-α: tumor necrosis factor α; VCAM-1: vascular cellular adhesion molecule-1; vmPFC: ventromedial prefrontal cortex; vWF: von Willibrand Factor

An established risk factor for ischemic heart disease [2, 26, 27], PTSD is associated with impaired hypothalamic-pituitary-adrenal (HPA**)** axis and hyperactivity of the sympathetic and renin-angiotensin systems. These responses provoke hypertension, dyslipidemia, chronic inflammation and a prothrombotic state, the “connectors” linking PTSD to atherosclerosis and its cardiovascular sequelae, e.g. ischemic heart disease, stroke and hypertension (Fig. 1).

## Atherosclerosis: Pathogenesis and Detection

Atherosclerosis, a chronic inflammatory disease of large- and medium-sized arteries characterized by the intima-medial accumulation of lipids, fibrous elements, inflammatory macrophages, lipid-engorged foam cells and cellular debris [28], is the predominant cause of cardiovascular ischemic disorders including stroke, myocardial infarction (MI) and peripheral artery disease [5]. The product of a complex and insidious pathological process, atherosclerosis progresses over a period of years, and its initial stages are symptomatically silent.

Diagnosis of atherosclerosis relies on imaging to detect vascular injury, and assessment of vascular stiffness using pulse wave velocity or ultrasonography. Angiography detects luminal narrowing and vascular wall lesions, and is best suited for evaluating advanced atherosclerosis, while analyses of coronary artery calcification and peripheral arterial stiffness detect nascent atherosclerosis and assess risk of atherosclerosis [4]. Arterial stiffness is a hallmark of atherosclerosis and vascular aging [29]. Healthy arterial walls are elastic, compliant and accommodating of pulsatile blood flow. As atherosclerosis progresses, collagen deposition, calcification and fibrous plaque formation stiffen arteries and reduce their elasticity.

Endothelial cells regulate vascular tone, control permeability, and modulate inflammation [5], while endothelial impairment augurs heightened risk of cardiovascular disease and mortality [30]. Endothelial injury or dysfunction, combined with smoking, hypertension, dyslipidemia, inflammation and other risk factors, allows low density lipoprotein particles to accumulate within the vascular intima, forming an atheroma. Vesicles generated from membranes of platelets and anti-inflammatory M2 macrophages delivered an endothelial-mesenchymal transition inhibitor and siRNA directed against the pro-atherosclerotic kinase PIM1 to injured endothelium in atherosclerotic plaques attenuated plaque progression by suppressing endothelial-mesenchymal transition, foam cell formation and inflammation within the lesions [31].

Endothelium, vascular smooth muscle cells and invading monocytes and macrophages release reactive oxygen species (ROS) which oxidize the accumulating lipids and trigger proinflammatory signaling that intensifies endothelial injury and atheroma development [32, 33]. In a single prolonged stress model of PTSD in mice, 21 days oral administration of the dietary supplement silymarin reversed deficits in working memory, locomotor activity and recognition, lowered malondialdehyde and nitrite contents, increased glutathione and antioxidant enzyme activities and preserved neuronal architecture in PTSD-susceptible prefrontal cortex, hippocampus and striatum [34].

By depleting tetrahydrobiopterin, an essential cofactor for endothelial nitric oxide (NO) synthase, ROS impair endothelial production of NO [35], the central mediator of flow-induced vasodilation and a suppressor of platelet aggregation, leukocyte adhesion, oxidative stress and smooth muscle proliferation. Reduced NO production and flow-mediated vasodilation characterize endothelial dysfunction [36]; consequently, military veterans with PTSD had impaired brachial artery flow-mediated vasodilation ascribable to decreased endothelial NO production [37]. A recent report demonstrated attenuated brachial flow velocity response to mental stress in premenopausal women with vs. without PTSD [38], confirming PTSD-associated endothelial dysfunction.

## Neuroendocrine PTSD-Atherosclerosis Connectors

Acute trauma mobilizes a complex neuroendocrine response encompassing the sympathetic nervous system, renin-angiotensin-vasopressin cascade and hypothalamic-pituitary-adrenocortical (HPA) axis [39]. Dysregulation of the neuroendocrine stress system engenders a maladaptive condition with unresolved stress activation and chronic elevation of mediators comprising an *allostatic load* [21]. Low serum cortisol and exaggerated catecholamine responses to stimuli are PTSD hallmarks and atherosclerosis risk factors [40]. These myriad neuroendocrine factors (Fig. 1) may be termed *PTSD-atherosclerosis connectors*, analogous to the cardiorenal connectors mediating the cardiorenal syndromes. Table 1 lists several clinical studies addressing PTSD – atherosclerosis relationships and their potential mechanisms.

**Table 1:** Clinical trials examining PTSD – atherosclerosis associations

Author(s), Year	Study Design	Assessment(s)	Main Outcomes
Grenon et al., 2016 [37]	Male veterans: 67 PTSD-positive, 147 PTSD-negative	Brachial artery flow-mediated vasodilation	PTSD independently associated with impaired flow-mediated vasodilation
Lima et al., 2020 [26]	Follow-up assessment of adult myocardial infarct (MI) survivors	Nuclear myocardial perfusion imaging	PTSD independently associated with increased incidence of mental stress-induced MI
Robicsek et al., 2011 [41]	Trauma-exposed patients: 30 PTSD-positive, 30 PTSD-negative	Systemic venous activities of von Willibrand factor (vWF) and clotting factor VIII	Positive association between PTSD severity and coagulation factor activities
Shkreli et al., 2026 [18]	Healthy adults underwent psychosocial stress after taking angiotensin receptor blocker losartan (*n* = 27) or placebo (*n* = 29)	Heart rate (HR), HR variability, subjective distress ratings, salivary cortisol activity	Single losartan dosage (50 mg *po*) did not alter responses to subsequent acute psychosocial stress
Tahmin et al., 2024 [38]	Premenopausal women: 21 PTSD-positive, 21 PTSD-free controls	Arterial blood pressure, HR, brachial artery blood flow velocity	Blood flow velocity increased in controls, decreased in PTSD-positive women during acute mental stress
Ten Brink et al., 2026 [42]	Coronary artery disease patients (*n* = 116; 61.4 ± 11.5 years) experiencing acute coronary syndrome	Continuous ambulatory HR and HR variability	Greater PTSD severity was associated with higher resting HR and decreased HR variability
Von Känel et al., 2006 [43]	Trauma-exposed subjects: 14 with, 14 without PTSD	Systemic venous activities of vWF, clotting factors	PTSD severity correlated with factor VIII activity
Von Känel et al., 2010 [44]	Post-MI patients: 23 with, 21 without PTSD	Plasma fibrinogen and D-dimer measured before and after trauma-specific interview	Trauma-specific interview provoked greater increases in fibrinogen and D-dimer in PTSD vs. non-PTSD patients
Yusuf et al., 2004 [45]	International trial in adults ≥ 40 years: 15,152 acute MI cases, 14,820 controls	Abdominal obesity, alcohol consumption, smoking, diabetes, hypertension, psychosocial factors	Chronic psychosocial stress increased odds of MI 2.67-fold

*HR*: heart rate; *MI*: myocardial infarction; *vWF*: von Willibrand factor

How PTSD, a disorder defined by psychological and neurological dysregulation promotes plaque initiation, progression, and destabilization can be understood as a convergence of multiple interacting maladaptive mechanisms (Fig. 1). The PTSD-atherosclerosis connectors arise as products of cortico-limbic dysregulation. Disruption of the uncinate fasciculus tract impaired communication between the amygdala and ventromedial prefrontal cortex, paralleling atherosclerosis of the aorta and carotid arteries in PTSD patients with stress-associated amygdala hyperactivity [6]. The amygdala-driven stress response constituting the PTSD-imposed allostatic load accelerates atherosclerosis and cardiovascular damage [21]. PTSD elicits neuronal co-expression of C-X-C chemokine receptor type 4 (CXCR4) and G protein subunit γ4 (GNG4), which form stable complexes in frontal cortical neurons [46]. Upregulation of CXCR4 and GNG4 correlated directly with intima-media thickness and inversely with lumen diameter in abdominal aorta of mice subjected to chronic stress [46]. Serum contents of CXCR4, GNG4, the CXCR4 ligand CXCL12, and pro-inflammatory IL-6 and IL-1β also were elevated in those mice [46]. By transmitting the central stress response to the periphery, these blood-borne mediators amplify systemic inflammation and atherogenesis.

### Autonomic Imbalance

Sympathetic hyperactivity provokes atherogenesis by stimulating mitochondrial ROS formation in cells within the vessel wall. Mitochondrial ROS overproduction impairs oxidative metabolism, depleting the vascular endothelium, smooth muscle, pericytes and macrophages of ATP for managing intracellular Ca^2+^, controlling macrophage polarization and maintaining arterial barrier integrity [47]. A study in apolipoprotein E^−/−^ mice, whose atherosclerosis risk is elevated, underscored the pivotal role of sympathetic hyperactivity in driving atherogenesis [48]. Increases in circulating norepinephrine and monoamine oxidase A activity were associated with ROS accumulation and damaged endothelial mitochondria in the apolipoprotein E^−/−^ mice, while renal sympathectomy attenuated mitochondrial ROS formation, atherosclerotic plaque burden, endothelial injury and vascular inflammation [48].

Catecholamine effects on monocytes connect the sympathetic nervous system to innate immunity. Dopamine upregulates monocyte expression of C-C chemokine receptor type 2 (CCR2), a chemokine receptor that recruits monocytes to arterial plaques [49]. In an innovative study of mechanisms linking social determinants of health to cardiovascular risk, Baumer et al. exposed monocytes from healthy individuals to serum with elevated dopamine and norepinephrine concentrations obtained from residents of socioeconomically deprived neighborhoods. The exposure increased monocyte CCR2 content, an effect recapitulated by incubation with dopamine but not norepinephrine [49].

In a study of PTSD and atherosclerotic risk factors in young adults, autonomic balance and aortic stiffness were evaluated by power spectral analysis of heart rate variability and measurements of carotid-to-femoral pulse wave velocity, respectively [29]. PTSD civilian checklist scores positively correlated with low-frequency/high-frequency power spectral ratios indicating adrenergic hyperactivity and autonomic imbalance. Pulse wave velocities revealed sex differences: PTSD score positively correlated with pulse wave velocity in the male subjects, suggesting increased aortic stiffness and sympathovagal imbalance may elevate cardiovascular disease risk in men with PTSD, while in the females the correlation was negative, suggesting reduced vasomotor function may be a cardiovascular risk factor in women with PTSD.

### Renin-Angiotensin System

A pivotal mediator of the stress response, the renin-angiotensin system (RAS) is another connector linking PTSD to inflammation and cardiovascular injury. During severe stress, sympathoadrenal activity and neuroinflammatory cytokines upregulate the classic angiotensin-converting enzyme (ACE) - angiotensin II (Ang II) – angiotensin type 1 (AT_1_) receptor axis [10].

Activation of RAS in rodents potentiates stress-related cardiovascular, endocrine, and behavioral responses across multiple stress paradigms. Rats modeling PTSD after protracted exposure to predator urine demonstrated enhanced hypertensive responses to a low Ang II dose and increased formation of proinflammatory cytokines and RAS pathway components [50]. In rats with lipopolysaccharide-induced inflammation, the AT_1_ receptor blocker candesartan lowered circulating pro-inflammatory cytokines and blunted expression of adhesion molecules in the paraventricular hypothalamic nucleus, subfornical organ, prefrontal cortex, hippocampus and amygdala, implicating RAS in the PTSD neuroinflammatory state [51]. Interestingly, mice subjected to Pavlovian fear conditioning demonstrated fear attenuation by the AT_2_ receptor isoform expressed in the medial central amygdala, where injection of AT_2_ receptor agonist C21 decreased fear-related freezing behavior [52].

Angiotensin II may interfere with consolidation of fear extinction memory in PTSD patients [53]. In healthy volunteers submitted to psychosocial stress, the AT_1_ receptor antagonist losartan attenuated PTSD-related disruption of the functional connectivity of the memory formation network (hippocampus and parahippocampal gyrus), without affecting the fear processing network (amygdala and insula) [18]. In female PTSD patients undergoing fear conditioning, low serum estradiol was associated with increased plasma renin activity and augmented tachycardic and startle responses [54]; thus, peri- and post-menopausal women with PTSD may be at heightened RAS-associated cardiovascular risk.

### Macrophage M1 Polarization and Foam Cells

PTSD activates multiple immune pathways that engage the atherogenic machinery. Chronic stress provokes release of pro-inflammatory cytokines IL-1β, IL-6, and TNF-ɑ, which drive macrophage transformation into foam cells. In a mouse PTSD model, 10 weeks of chronic unpredictable stress raised serum IL-1β and lipids, disrupted the intimal layer of the thoracic aorta, and increased macrophage membrane content of the oxidized lipoprotein receptor, CD68 [55], implicating the PTSD-induced inflammatory cascade in stress-induced atherogenesis. In PTSD patients, increased ^18^F-deoxyglucose uptake in the spleen and bone marrow and cardiac-specific high-sensitivity C-reactive protein also indicated hematopoietic activation [6]. The accelerated leucopoiesis and monocyte release relates cortico-limbic neural activity to an immune cascade adversely impacting the cardiovascular system

Vascular cell adhesion molecule-1 and intercellular adhesion molecule-1 mediate monocyte-endothelial adhesion. Adherent monocytes invade atherosclerotic plaques [56] where they differentiate into macrophages which assume two distinct polarities, M1 or M2 [57]. M1 macrophages predominate in the rupture-prone shoulders of atherosclerotic plaques [58]. Oxidized low-density lipoproteins in the atheroma cause M1 macrophages to shift from long chain fatty acid catabolism to glycolysis [59]; consequently, M1 macrophages accumulate lipid droplets and transform into lipid-laden foam cells [58]. Foam cells generate ROS and cytokines to sustain inflammation [58], while anti-inflammatory M2 macrophages promote tissue repair. PTSD is associated with increased vascular ROS production [60]. Elevated intracellular cholesterol may cause visceral smooth muscle cells to transdifferentiate into foam cells [61, 62]. Plaques with thin fibrous caps are particularly vulnerable to rupture [63], initiating platelet activation and thrombosis that narrows or occludes the arterial lumen, culminating in MI or stroke. PTSD is associated with elevated serum triglycerides, low-density lipoproteins and cholesterol, regardless of patient age, body mass index or medications [64].

### Hypothalamo-Pituitary-Adrenocortical Axis and Cortisol

Subnormal circulating cortisol activity may contribute to the progression of atherosclerosis by permitting endothelial production of cytokines which chemoattract monocytes to the developing lesion [65, 66]. In adult offspring of Holocaust survivors, those with PTSD had decreased urinary cortisol excretion [67]. Similarly, among 172 adult survivors of traumatic injury, those with low serum cortisol 2–3 days post-trauma developed severe PTSD 6–8 months later, while patients with initially high cortisol developed dysphoria instead [68]. A meta-analysis of clinical studies assessing plasma or serum cortisol concentrations, 4 exclusively in women and 10 exclusively in men, identified significantly lower cortisol in women with vs. without PTSD, but no significant PTSD association in men [69]

### Systemic and Intravascular Inflammation

The PTSD-associated proinflammatory state provides a mechanistic bridge to atherosclerosis, an inflammatory disease of the arterial wall. Sympathoadrenal hyperactivation and elevated pro- vs. anti-inflammatory T-lymphocyte ratio increase circulating pro-inflammatory cytokines and the inflammation biomarker, C-reactive protein, causing dysfunctional neuroendocrine-immune interactions [7]. Reduced glucocorticoid tone in individuals with PTSD limits cortisol suppression of cytokine production. Repeated restraint stress in rats increased interleukin-1β (IL-1β) and inducible NO synthase in the prefrontal cortex, hippocampus, and hypothalamus, and disrupted the IL-1β-driven adrenocorticotropic hormone and corticosterone secretory response to acute stress [70]. The glucocorticoid suppression suggests that the HPA dysregulation and inflammatory processes are not static but evolve over the duration of chronic stress and provoke cardiovascular complications of varying severities and time courses. Chronic stress also drives adrenal hypertrophy and amplifies adrenal glucocorticoid and catecholamine release [60]. These neuroanatomical and neurochemical changes underscore the urgent need to identify cardiac biomarkers in PTSD patients as indicators of cardiovascular disease and treatment efficacy [71]

Inflammation is a fundamental catalyst for atherosclerosis. Endothelial injury from oxidant overproduction activates macrophages, lymphocytes and endothelial cells to release tumor necrosis factor-α (TNF-α), IL-1β and interleukin-6 (IL-6) [72, 73]. These cytokines intensify inflammation by recruiting leukocytes, activating endothelial cells and provoking vascular smooth muscle proliferation. PTSD is associated with an exaggerated inflammatory response with elevated circulating TNF-α, IL-1β and IL-6; thus, inflammation is a pathogenic mechanism common to atherosclerosis and PTSD [60].

The phosphatidylinositol-3 kinase (PI3K)/Akt/mechanistic target of rapamycin (mTOR) signaling cascade is a master regulator of vascular metabolism, inflammation, and endothelial function [74] and centrally, of synaptic plasticity, memory consolidation and satiety [75]. In metabolic disorders, PI3K/Akt/mTOR hyperactivation by mitogen-activated protein kinase produces oxidative stress, endothelial dysfunction and atherogenesis [74]. In PTSD, dysregulated mTOR signaling in the limbic system and prefrontal cortex is implicated in abnormal fear extinction, maladaptive synaptic hyperplasticity, and metabolic dysregulation predisposing to obesity [75]. PI3K/Akt/mTOR signaling controls macrophage survival and cytokine production, but its overactivation promotes macrophage M1 polarization and TNF-α, IL-1β and IL-6 release, exacerbating vascular inflammation and progression of atherosclerosis [74].

### Circulating Lipids

Alterations in blood composition effect the interface between PTSD neurobiological state and accelerated arterial atherogenesis. PTSD dysregulates lipid metabolism and elevates low density lipoproteins, cholesterol and triglycerides, which promotes lipid loading in the atheroma [5]. Elevated lipids in mice subjected to chronic unpredictable stress promoted plaque deposition and fibrosis in the thoracic aorta [55], establishing a direct link between stress-driven hyperlipidemia and atherogenesis. Chronic stress also is associated with accumulation of visceral adipose tissue which increases inflammatory markers and ignites adipose-driven atherogenic inflammation [76]. In macrophages, chronic stress hyperactivates AMPK/mTOR signaling, promotes lipid accumulation and impairs cholesterol efflux, driving macrophage transformation into foam cells [74].

### Clotting Cascade and Hypercoagulability

Pro-thrombotic states augur increased risk of atherosclerotic disease and poor prognosis. Acute or chronic stress elicits hypercoagulability. Likely mediators include sympathetic hyperactivity, vagal withdrawal, the HPA axis, and elevated circulating catecholamines and vasopressin [77]. These factors interact with endothelial dysfunction, inflammation, thrombopoiesis and hemoconcentration to instigate hypercoagulability. Myocardial infarction survivors with PTSD demonstrated elevated serum fibrinogen and D-dimer levels and heightened pro-coagulative reactivity vs. non-PTSD survivors, increasing the risk of thrombosis exacerbating cardiovascular disease [44].

Hypercoagulability mechanistically connects PTSD and atherothrombosis. Concordant with the allostatic load model, the hypercoagulable state is initially adaptive to acute stress but becomes maladaptive with excessive or prolonged stress [77]. PTSD hyperarousal elevates circulating clotting factor VIII, which exerts its procoagulant activity by protecting circulating von Willibrand factor (vWF), and fibrinogen, a coagulation cascade substrate that promotes platelet aggregation [43]. These relationships also accompanied subthreshold PTSD symptoms, suggesting that hypercoagulability exists across the PTSD severity spectrum. Patients with severe, protracted PTSD also demonstrate elevated plasma vWF and factor VIII contents [41]. These circulating factors link PTSD to atherosclerotic vascular damage.

### Metabolic Derangements

Due to the dysregulation of the HPA and sympathoadrenal systems, PTSD patients are at heightened risk for insulin-resistance and dyslipidemia [39]. Abnormal lipid metabolism is an atherosclerosis hallmark [74]. Dysregulation of lipid trafficking in both PTSD and atherosclerosis promotes lipid accumulation in the developing atheroma. Heightened stress also is associated with greater visceral and subcutaneous adipose tissue, leading to increased adiposity-related inflammation [76]. Chronic stress alters the blood lipid composition such that circulating concentrations of low density lipoproteins, cholesterol, and triglycerides increase significantly, raising the atherosclerosis index [5]. The mechanisms of deranged lipid metabolism, such as adipose tissue lipolysis that provides the substrates for plaque formation, further accelerate lipid-driven atherosclerosis.

## Relationship of PTSD to Atherosclerotic Pathogenesis

Chronic stress and psychiatric disorders, including PTSD, are established atherosclerosis risk factors, but a direct role of these disorders in atherosclerosis causation is a matter of debate. While some studies reported positive associations between PTSD and atherosclerotic markers, others yielded null findings that question the nature of the relationship. This ambiguity necessitates a critical analysis of the available evidence with particular attention to the subject populations, study design, atherosclerotic outcome, and endpoints.

### Evidence in Humans that PTSD Predisposes to Atherosclerosis

A multi-center clinical trial in 15,152 adults with MI vs. 14,820 MI-free controls without demonstrated chronic psychosocial stress (stress at work or home, financial stress, adverse life events, depression) to be associated with 2.67-fold increased likelihood of MI [45]. A potential cause-and-effect relationship between the consequences of combat stress and atherosclerosis, leading to accelerated biological aging, has been reported [78]. Additionally, atherosclerosis can affect neurocognitive function by compromising cerebral perfusion. Of 125 military veterans (73.4 ± 6.7 years of age) receiving treatment for peripheral arteriosclerosis, 61% had cognitive impairment, and PTSD was more prevalent in the impaired subjects than their cognitively intact counterparts (80% vs. 57%; *P* = 0.02) [79].

In veterans without known coronary artery disease, those with PTSD had a greater coronary arterial calcified plaque burden than those without PTSD, and the coronary artery calcification (CAC) scores were significantly higher for the PTSD patients in each Framingham risk score category [4]. After adjustment for cardiovascular risk factors, CAC and PTSD were independent predictors of mortality; PTSD increased the risk of death by nearly 2.3-fold, linking PTSD to cardiovascular mortality through atherosclerotic burden. Subsequent work demonstrated mild traumatic brain injury (mTBI) to be an independent predictor of atherosclerosis severity and CAC, yet combined mTBI and PTSD provided more reliable prognosis of cardiovascular mortality than mTBI alone [80]. A recent study identified aortic CAC score as an independent mortality predictor in mTBI/PTSD patients with heightened carotid and aortic atherosclerotic burden and cortico-limbic disruption [6]. These reports suggest that combined PTSD and mTBI may amplify atherosclerosis and the attendant mortality risk.

Carotid intima media thickness (CIMT) is another marker for subclinical atherosclerosis and cardiovascular disease prognosis. A rigorous study in 465 sets of twin Vietnam veterans where one had PTSD demonstrated a statistically significant association between PTSD and CIMT, where CIMT was 33 μm greater in the twins with PTSD [9]. Combat exposure demonstrated a similar association with CIMT, but was no longer an independent CIMT predictor when PTSD was included, suggesting vulnerability to PTSD had the greater influence. Recently, PTSD symptom intensity was found to be associated with greater CIMT and carotid atherosclerosis in community-based midlife women [8], a population with comparatively less trauma burden and cardiovascular risk than combat veterans. The adverse cardiovascular and neurocognitive sequelae in these women indicates that the adverse PTSD – atherosclerosis relationship is not confined to high-risk military groups.

### Evidence in Humans that PTSD Does not Increase Atherosclerosis Risk

The evidence for PTSD causation of atherosclerosis is by no means unequivocal. A recent study of twin Vietnam veterans revealed a non-significant − 0.95% difference in CAC scores per increment in PTSD symptoms, despite the inherent matching of familial and early environmental factors [10]. The non-significant association and rigorous study design isolating the independent effect of PTSD from shared vulnerability factors, strongly suggests PTSD is not a direct cause of coronary atherosclerosis.

Another study assessed carotid artery plaque and intima-media thickening by ultrasonography in 3119 men and women, 54.5% of whom had a history of trauma exposure, but only 2% had PTSD [81]. After correcting for cardiovascular disease risk factors and other covariates, and stratifying results into five age ranges (< 40, 40–49, 50–59, 60–69 and > 70 years), those who experienced trauma (with or without PTSD) had increased odds of self-reported cardiovascular events (odds ratio 1.51; 95% CI 1.03–2.22), but only the 40–49 year olds showed a statistically significant correlation between CIMT and trauma ± PTSD. Since this study was not limited to veterans, the findings suggest that positive findings may be selective for veterans, and population-specific factors may have a larger role in atherosclerosis.

The twin Vietnam veterans study also analyzed within-pairs, or co-twins in each pair, and showed no significant differences in CIMT when comparing PTSD-discordant co-twins; thus, familial or genetic factors may be stronger CIMT determinants than PTSD or combat exposure [9]. These findings imply that PTSD may be more associated with acute coronary events and myocardial ischemia than with whole-body atherosclerotic risk factors, e.g. hypertension and dyslipidemia. Neither atherosclerotic inflammation nor carotid arteriosclerotic burden differed in trauma survivors with vs. without PTSD [6], suggesting that PTSD does not exacerbate positron emission tomography-detectable plaque inflammation.

### Potential Explanations for Divergent PTSD – Atherosclerosis Relationships

Whether the PTSD – atherosclerosis relationship represents cause-and-effect vs. co-phenomena remains unresolved. The divergent reports of positive PTSD-atherosclerosis associations vs. null findings reflects the mechanistic complexity and multiple interacting factors (Fig. 1) shaping the relationship. Several explanatory frameworks may help reconcile the divergent findings. The twin studies [5, 7, 10] show how common genetic or early developmental vulnerabilities increase susceptibility to both PTSD and atherosclerosis even without one disorder causing the other. Although adjusting for confounding variables weakens the PTSD-atherosclerosis association, PTSD manifests as other cardiovascular complications. A salient question is which populations to study. The majority of positive studies were conducted on military veterans, who carry a high burden of PTSD and other comorbidities. The positive PTSD – atherosclerosis association in midlife women [8] suggests that non-veteran PTSD populations also are predisposed to develop atherosclerosis.

The different methodologies for evaluating PTSD and cardiovascular diseases can contribute to outcome heterogeneities. Combat exposure typically serves as a proxy for PTSD and, consequently, studies may forgo a formal psychiatric assessment aligned with the DSM-5 guidelines [1]. Other assessments of vascular function and CVD, e.g. endothelial dysfunction and arterial stiffness, have been underutilized. A broader methodological selection is needed since the effects of PTSD on the arterial system may be anatomically heterogenous given the dynamic physiology of cardiac output distribution, e.g. during exercise, digestion or ascent to altitude. Analysis of the effects of structural remodeling versus inflammation during acute or chronic stress may reveal diverse cardiovascular consequences.

## PTSD and Atherosclerosis: Sequential, Parallel or Bidirectional Pathogenesis?

**Fig. 2:**
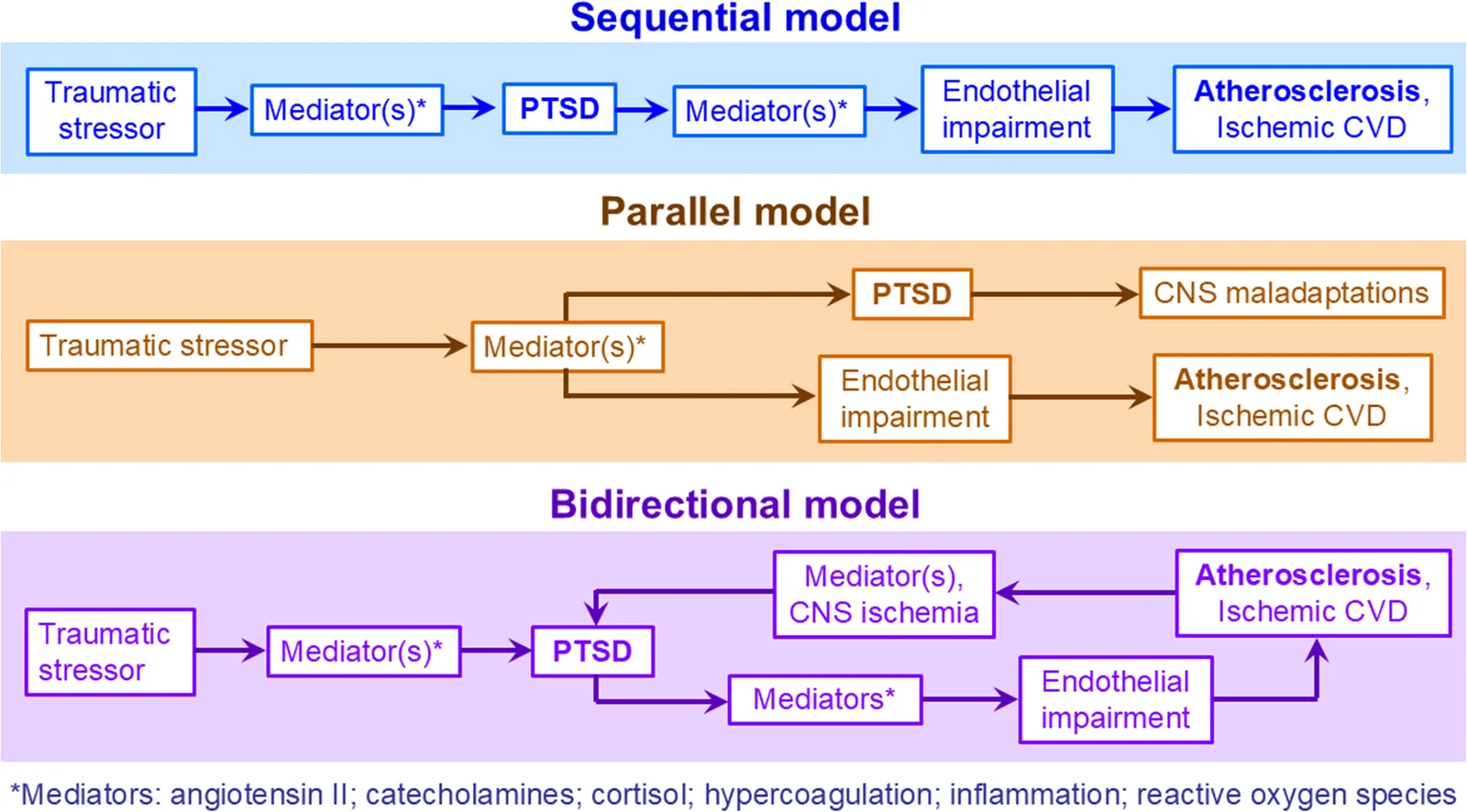
Models of PTSD – atherosclerosis relationships. The sequential model proposes a linear progression from acute trauma to PTSD to endothelial impairment to atherosclerosis, mediated by neuroendocrine connectors. In the parallel model, trauma-activated mediators elicit PTSD and vascular injury independently. The bidirectional model incorporates PTSD induction by atherosclerosis and its consequences into the sequential model, effecting a feed-forward cycle. In the bidirectional model, some mediators of the atherosclerosis → PTSD reverse pathway may differ from those mediating the trauma → PTSD → endothelium → atherosclerosis pathway. CNS: central nervous system; CVD: cardiovascular disease

Understanding the nature of the PTSD-atherosclerosis relationship is crucial to inform development of treatments for one or both disorders. Associations between PTSD and atherosclerosis can be categorized as *sequential*, *parallel* and *bidirectional* (Fig. 2). These patterns are not mutually exclusive, and their respective contributions may depend on the pathological context.

### Sequential Model: PTSD as a Driver of Atherosclerosis

The sequential model (Fig. 2) posits that PTSD is a mechanistic predecessor of atherosclerosis, wherein PTSD-induced adrenergic hyperactivity, RAS upregulation, proinflammatory cytokine release, endothelial dysfunction, and/or hypercoagulability provoke atherosclerosis [5, 48, 50, 77]. Substantial empirical evidence supports this model. For example, predatory stress-induced RAS activation and serum proinflammatory cytokines predicted hypertensive responses to slow-pressor Ang II dosages in rats, showing that PTSD-induced mediators can lower the threshold of cardiovascular pathogenesis [50]. A recent analysis of nearly 400,000 female veterans found that women with PTSD were 50% more likely to experience ischemic heart disease than their non-PTSD counterparts, with the greatest disparity in the youngest (40–49 years old) age group [2]. Similarly, a study of nearly 1 million veterans (mean age 30 years), 28% of whom had PTSD, demonstrated a 31% increase in cumulative atrial fibrillation incidence in male and female PTSD vs. non-PTSD veterans [82], suggesting that the autonomic imbalance can predispose PTSD patients to cardiac arrhythmias.

### Parallel Model

The parallel model (Fig. 2) proposes that PTSD and atherosclerosis arise from common etiologies, rather than one condition causing the other. According to this model, PTSD and atherosclerosis share common factors that mediate independent progression of both disorders.

Goetz et al. [9] and Correia et al. [10] examined associations between atherosclerotic burden and PTSD within pairs of twins with shared genetic background, environment, and socioeconomic factors. Although PTSD was associated with cardiovascular disease burden, neither study demonstrated significant PTSD-associated changes in CAC or CIMT, arguing against a direct PTSD – atherosclerosis sequential relationship in favor of a parallel one.

A recent genome wide association study of over 700,000 patients with PTSD, Major Depressive Disorder and anxiety disorders identified genetic correlations with premature mortality from cardiovascular diseases, specifically coronary atherosclerosis, hypertension and major coronary artery disease [83]. Several abnormal genes and/or pathways which heightened the risk for mood disorders also increased the risk of atherosclerosis and related cardiovascular diseases in this large cohort. This study established a genetic basis for parallel susceptibility to atherosclerotic disease vs. PTSD and similar disorders.

### Integrative Model: Bidirectional Amplification

In some cases a reverse atherosclerosis - PTSD relationship may develop, where the trauma of a life-threatening cardiac event, e.g. sudden cardiac arrest or MI produces persistent fear of recurrence in patients and/or their caregivers [42, 84]. The bidirectional model (Fig. 2) embodies the concept of *shared vulnerability*, in which environmental and developmental exposures, chronic stressors and genetic variants can predispose patients to both PTSD and cardiovascular burden. The forward (PTSD → atherosclerosis) and reverse (atherosclerosis → PTSD) relationships may have different mediators [9, 10, 83].

Stroke, MI and other atherosclerotic events can provoke PTSD, which in turn may trigger inflammation, procoagulant responses, and autonomic hyperactivity that exacerbate vascular disease [77]. This maladaptive feedforward cycle would progressively amplify both vascular disease and PTSD [2], increasing the risk of recurrence of cardiovascular events [42] and, consequently, mortality [80]. The bidirectional model identifies PTSD as a modifiable atherosclerosis risk factor and informs treatment of the associated cardiovascular disease. Thus, screening for both PTSD and atherosclerosis, and analysis of the genetic context may guide efforts to optimize atherosclerotic disease treatment in PTSD patients.

### Clinical Implications of the Models

The well-documented concomitance of PTSD and atherosclerotic burden argues for changes in clinical practice, whether the two phenomena are linked by a cause-and-effect relationship, or not. PTSD imposes a cardiovascular risk profile, and clinicians managing PTSD patients should incorporate structured risk assessments as routine components of longitudinal care, as is done in patients with established risk-amplifying comorbidities. The bidirectional model demonstrates how cardiac events provoke PTSD, which intensifies procoagulant reactivity to stress and worsens thrombotic risk [5]. Thus, PTSD screening in the weeks following a cardiac event is a practical means of assessing risk of recurrent thrombosis.

Additional potential treatments stem from the cardiovascular indication for antihypertension therapy in PTSD patients. As mainstay interventions for many cardiovascular indications, ACE-inhibitors and angiotensin receptor blockers emerge as options to promote fear extinction and limit PTSD-driven complications including neuroinflammation and hypertension [10]. Quelling PTSD symptoms could interrupt pathogenesis of atherosclerosis and promote vascular repair. Grenon et al. identified a treatment disparity, wherein PTSD patients are less likely to receive ACE-inhibitors (17% versus 36%) or beta-blocker treatment (25% versus 41%) than their non-PTSD counterparts [37]. Thus, early PTSD diagnosis could enable early detection and treatment of nascent atherosclerosis.

### Novel Interventions for Atherosclerosis and PTSD

Recent studies in mice evaluated novel interventions for atherosclerosis. Vesicles generated from membranes of platelets and anti-inflammatory M2 macrophages delivered an endothelial-mesenchymal transition inhibitor and siRNA directed against the pro-atherosclerotic kinase PIM1 to injured endothelium in atherosclerotic plaques attenuated plaque progression by suppressing endothelial-mesenchymal transition, foam cell formation and inflammation within the lesions [31]. In a single prolonged stress model of PTSD in mice, 21 days oral administration of silymarin reversed deficits in working memory, locomotor activity and recognition, lowered malondialdehyde and nitrite contents, increased glutathione and antioxidant enzyme activities and preserved neuronal architecture in PTSD-susceptible prefrontal cortex, hippocampus and striatum [34]. In rabbits fed a high fat diet to model atherosclerosis, the natural bioactive plant alkaloid rhynchophylline suppressed oxidized low-density lipoprotein uptake, foam cell formation, endothelial expression of adhesion molecules and transendothelial migration of monocytes [85]. Rhynchophylline also suppressed mitogen activated protein kinase and nuclear factor-κB signaling cascades, thereby attenuating ROS formation and inflammation, pivotal pathogenic mediators of atherosclerosis. The potential of rhynchophylline to limit the pathogenesis of PTSD merits investigation.

### Limitations

Lifetime PTSD prevalence is approximately threefold greater in women than men [17], yet many clinical studies of the PTSD - atherosclerosis association were conducted in military veterans, a predominantly male population [3, 15, 79]. Although several recent studies were conducted in women [8, 38, 54, 81], there remains a need for PTSD – atherosclerosis clinical studies with equal representation of both sexes, and in postmenopausal women whose diminished circulating estradiol increases their vulnerability to renin-angiotensin hyperactivation [54]. The divergent PTSD-vascular stiffness relationships in men, in whom PTSD was associated with increased pulse wave velocity, vs. women, in whom PTSD was associated with decreased pulse wave velocity [29], underscores this critical need.

### Knowledge Gaps

Preclinical and clinical studies (Table 1) have defined several mechanisms common to PTSD and atherosclerosis that may contribute to the pathogenesis of both (Fig. 1). However, the relative impacts of these mechanisms on PTSD and atherosclerosis are undefined, and may vary with the etiology, severity and/or duration of one or both conditions. Indeed, some mechanisms may contribute to sequential or bidirectional co-development of PTSD and atherosclerosis, such that interventions targeting those mechanisms may prove efficacious against both conditions, while others may provoke PTSD and/or atherosclerosis in isolation.

### Future Directions

There is an urgent need for clinical trials examining treatments directed against both PTSD and atherosclerosis. Natural products including silymarin [34] and rhynchophylline [85] showed therapeutic benefit in animal models of PTSD [34] and atherosclerosis [85], respectively. The possibility that these and other natural products might prove effective against both PTSD and atherosclerosis remains to be tested in animal models and, ultimately, clinical trials.

## Conclusion

The complex relationship between PTSD and atherosclerotic cardiovascular disease has emerged as a clinically significant intersection of the fields of psychiatry and cardiovascular medicine. PTSD, itself the product of adrenergic, HPA and renin-angiotensin hyperactivation, provokes several mechanisms and factors implicated in atherogenesis including Ang II, catecholamines, dysregulation of the HPA and clotting cascades, oxidative stress and inflammation. Recent work has refined understanding of the parallel, sequential and bidirectional aspects of this comorbid relationship, although as yet these relationships are not fully resolved. Current evidence supports an integrative model incorporating a vulnerable psychobiological background and the potential for feedforward amplification of PTSD and atherosclerosis. A pivotal question is whether PTSD treatment, either pharmacological or psychotherapeutic, can delay or even prevent atherosclerosis. Robust evidence addressing these questions may inform efforts to translate the mechanistic insights into cardiovascular prevention and timely psychiatric intervention. Recognition of PTSD as a modifiable cardiovascular risk factor is imperative for the millions of PTSD patients worldwide.

## Key References

- Gharios C, van Leent MMT, Chang HL, Abohashem S, O’Connor D, Osborne MT, et al. Cortico-limbic interactions and carotid atherosclerotic burden during chronic stress exposure. Eur Heart J 2024;45:1753-1764. doi: 10.1093/eurheartj/ehae149.
  - ○ This report demonstrated heightened stress-associated neural network activity in PTSD patients, and provided neuroimaging evidence linking cortico-limbic dysregulation in PTSD to peripheral vascular pathology.
- Shkreli L, Morse L, Scaife J, Browning M, Murphy SE, Kirschbaum C, Reinecke A. The effect of acute angiotensin receptor blockade on subjective and biological markers of stress response. Psychoneuroendocrinology 2026;191:107944. doi: 10.1016/j.psyneuen.2026.107944.
  - ○ This clinical study implicated angiotensin II in PTSD-associated disruption of the central fear processing and memory formation circuitry.
- Emudainohwo JOT, Ben-Azu B, Esuku DT, Chijioke BS, Iwhiwhu P, Usin SG, et al. Silymarin reverses post-traumatic stress-induced memory impairment in mice by containment of oxidative stress, cholinergic dysfunction, and modulation of neuronal caspase-3 expression. Naunyn Schmiedebergs Arch Pharmacol 2026;399:16371-94. doi: 10.1007/s00210-026-05356-z.
  - ○ This analysis of large cohort and case-crossover studies revealed associations of PTSD and other emotional disorders with heightened risk of atherothrombotic and acute coronary diseases.
- Tahmin CI, Tahsin CT, Wattero R, Ahmed Z, Corbin C, Carter JR, et al. Blunted brachial blood flow velocity response to acute mental stress in PTSD females. Physiol Rep 2024;12:e16137. doi: 10.14814/phy2.16137.
  - ○ This important clinical study demonstrated PTSD to be associated with endothelial dysfunction in conduit arteries of premenopausal women.
- Wang Q, Meng L, Xu R, Yin J, Liu D, Xu L, et al. Relationship between CXCR4 and GNG4 in the brain and chronic stress-induced atherosclerosis. J Mol Med 2025;103:1113-34. doi: 10.1007/s00109-025-02572-7.
  - ○ This study in mice defined molecular mechanisms in the frontal cortex linking chronic stress to atherosclerosis.
- Baumer Y, Pita MA, Turner BS, Baez AS, Ortiz-Whittingham LR, Gutierrez-Huerta CA, et al. Neighborhood socioeconomic deprivation and individual-level socioeconomic status are associated with changes to dopamine-mediated changes to monocyte subset CCR2 expression via a cAMP dependent pathway. Brain Behav Immun Health 2023;30:100640. doi: 10.1016/j.bbih.2023.100640.
  - ○ This innovative study demonstrated how serum catecholamines link adverse socioeconomic conditions to increased monocyte infiltration of the developing atheroma.
- Tian Y, Ullah H, Gu J, Li K. Immune-metabolic mechanisms of post-traumatic stress disorder and atherosclerosis. Front Physiol 2023;14:1123692. doi: 10.3389/fphys.2023.1123692.
  - ○ This study implicated hyperactivation of mTOR signaling as a mechanism linking PTSD to induction of atherogenesis.
- Smith EJT, Gasper WJ, Schneider PA, Finlayson E, Walter LC, Covinsky KE, et al. Cognitive impairment is common in a Veterans Affairs population with peripheral artery disease. Ann Vasc Surg 2023;91:210-7. doi: 10.1016/j.avsg.2022.11.029.
  - ○ This study identified peripheral arterial disease as a late-stage atherosclerotic complication in military veterans with high PTSD incidence and underdiagnosed cognitive impairment.

## Data Availability

No datasets were generated or analysed during the current study.

## Abbreviations

ACE: Angiotensin converting enzyme
CAC: Coronary artery calcification
CIMT: Carotid intima media thickness
CXCR4: C-X-C chemokine receptor type 4
GNGR4: G protein subunit *γ*4
HPA: Hypothalamic-pituitary-adrenal
HR: Heart rate
IL: Interleukin
MI: Myocardial infarction
mTBI: Mild traumatic brain injury
mTOR: Mechanistic target of rapamycin
NO: Nitric oxide
PI3K: Phosphatidylinositol-3 kinase
PTSD: Post-traumatic stress disorder
RAS: Renin-angiotensin system
ROS: Reactive oxygen species
TNF-α: Tumor necrosis factor-α
vWF: Von Willibrand factor
